# A Metabolomic Severity Score for Airflow Obstruction and Emphysema

**DOI:** 10.3390/metabo12050368

**Published:** 2022-04-19

**Authors:** Suneeta Godbole, Wassim W. Labaki, Katherine A. Pratte, Andrew Hill, Matthew Moll, Annette T. Hastie, Stephen P. Peters, Andrew Gregory, Victor E. Ortega, Dawn DeMeo, Michael H. Cho, Surya P. Bhatt, J. Michael Wells, Igor Barjaktarevic, Kathleen A. Stringer, Alejandro Comellas, Wanda O’Neal, Katerina Kechris, Russell P. Bowler

**Affiliations:** 1 Department of Biostatistics and Informatics, Colorado School of Public Health, University of Colorado Anschutz Medical Campus, Aurora, CO 80045, USA; katerina.kechris@cuanschutz.edu; 2 Division of Pulmonary and Critical Care Medicine, University of Michigan, Ann Arbor, MI 48109, USA; wlabaki@med.umich.edu (W.W.L.); stringek@umich.edu (K.A.S.); 3 Division of Medicine, National Jewish Health, Denver, CO 80206, USA; prattek@njhealth.org (K.A.P.); hilla@njhealth.org (A.H.); bowlerr@njhealth.org (R.P.B.); 4 Channing Division of Pulmonary and Critical Care Medicine, Brigham and Women’s Hospital, Boston, MA 02115, USA; remol@channing.harvard.edu (M.M.); redld@channing.harvard.edu (D.D.); remhc@channing.harvard.edu (M.H.C.); 5 Channing Division of Network Medicine, Brigham and Women’s Hospital, Boston, MA 02115, USA; reang@channing.harvard.edu; 6 Section on Pulmonary, Critical Care, Allergy & Immunology, Internal Medicine, Wake Forest School of Medicine, Winston Salem, NC 27157, USA; ahastie@wakehealth.edu; 7 Section on Pulmonary, Critical Care, Allergy & Immunology, Internal Medicine, Atrium Health Wake Forest Baptist, Winston Salem, NC 20157, USA; sppeters@wakehealth.edu; 8 Division of Respiratory Medicine, Department of Internal Medicine, Center for Individualized Medicine, Mayo Clinic, Scottsdale, AZ 85259, USA; ortega.victor@mayo.edu; 9 Division of Pulmonary, Allergy and Critical Care Medicine, University of Alabama at Birmingham, Birmingham, AL 35294, USA; sbhatt@uabmc.edu; 10 UAB Lung Health Center, Division of Pulmonary, Allergy, and Critical Care Medicine, Department of Medicine, University of Alabama at Birmingham, Birmingham, AL 35294, USA; jmwells@uabmc.edu; 11 Division of Pulmonary and Critical Care, David Geffen School of Medicine, University of California, Los Angeles, CA 90095, USA; ibarjaktarevic@mednet.ucla.edu; 12 Department of Clinical Pharmacy and the NMR Metabolomics Laboratory, College of Pharmacy, University of Michigan, Ann Arbor, MI 48109, USA; 13 Division of Pulmonary and Critical Care, University of Iowa, Iowa City, IA 52242, USA; alejandro-comellas@uiowa.edu; 14 Marsico Lung Institute, University of North Carolina, Chapel Hill, NC 27599, USA

**Keywords:** metabolomics, COPD, lung density, adaptive LASSO

## Abstract

Chronic obstructive pulmonary disease (COPD) is a disease with marked metabolic disturbance. Previous studies have shown the association between single metabolites and lung function for COPD, but whether a combination of metabolites could predict phenotype is unknown. We developed metabolomic severity scores using plasma metabolomics from the Metabolon platform from two US cohorts of ever-smokers: the Subpopulations and Intermediate Outcome Measures in COPD Study (SPIROMICS) (*n* = 648; training/testing cohort; 72% non-Hispanic, white; average age 63 years) and the COPDGene Study (*n* = 1120; validation cohort; 92% non-Hispanic, white; average age 67 years). Separate adaptive LASSO (adaLASSO) models were used to model forced expiratory volume at one second (FEV_1_) and MESA-adjusted lung density using 762 metabolites common between studies. Metabolite coefficients selected by the adaLASSO procedure were used to create a metabolomic severity score (metSS) for each outcome. A total of 132 metabolites were selected to create a metSS for FEV_1_. The metSS-only models explained 64.8% and 31.7% of the variability in FEV_1_ in the training and validation cohorts, respectively. For MESA-adjusted lung density, 129 metabolites were selected, and metSS-only models explained 59.0% of the variability in the training cohort and 17.4% in the validation cohort. Regression models including both clinical covariates and the metSS explained more variability than either the clinical covariate or metSS-only models (53.4% vs. 46.4% and 31.6%) in the validation dataset. The metabolomic pathways for arginine biosynthesis; aminoacyl-tRNA biosynthesis; and glycine, serine, and threonine pathway were enriched by adaLASSO metabolites for FEV_1_. This is the first demonstration of a respiratory metabolomic severity score, which shows how a metSS can add explanation of variance to clinical predictors of FEV_1_ and MESA-adjusted lung density. The advantage of a comprehensive metSS is that it explains more disease than individual metabolites and can account for substantial collinearity among classes of metabolites. Future studies should be performed to determine whether metSSs are similar in younger, and more racially and ethnically diverse populations as well as whether a metabolomic severity score can predict disease development in individuals who do not yet have COPD.

## 1. Introduction

Chronic obstructive pulmonary disease (COPD) is characterized by persistent respiratory symptoms and airflow limitation that is due to airway and/or alveolar abnormalities usually caused by significant exposure to noxious particles or gases [1]. COPD is one of the leading causes of death and hospitalizations worldwide and in the United States [2,3]. However, many adults with abnormal pulmonary function are not aware of having any obstructive lung disease [2]. Although the lung is the main affected organ, there is strong evidence for systemic effects of COPD as evidenced by muscle wasting, cardiovascular disease, osteoporosis, and depression, which suggests a generalized metabolic disturbance in affected individuals [2]. Metabolomic profiling of blood may help to assess these metabolic disturbances.

Several studies have examined the association between metabolites and lung function measures. In investigating forced expiratory volume in 1 s (FEV_1_), FEV_1_ as a percentage of predicted and the FEV_1_/Forced Vital Capacity (FVC) ratio, Cruickshank-Quinn et. al found 32 and 269 significantly associated metabolites, respectively, in plasma samples from 131 participants from a cohort with COPD [4]. They found significant associations between glycerophospholipid metabolism and FEV_1_ percent predicted, and between sphingolipids and FEV_1_/FVC [4]. In a large general population study (*n* = 4742), Yu et al. reported 30 novel metabolites associated with FEV_1_, out of a total of 95 associated metabolites with FEV_1_ using plasma samples [5]. Additionally, they found 100 metabolites associated with FVC. Yu et al. also showed associations between FEV_1_ and four metabolic pathways, including aminoacyl-tRNA biosynthesis; phenylalanine metabolism; nitrogen metabolism; and alanine, aspartate, and glutamate metabolism [5]. Kelly et al. found 156 metabolites associated with FEV_1_ in a general population study of 10,460 participants with a validation cohort of 437 participants using blood and plasma samples [6].

The association between COPD, metabolic pathways, and clusters of metabolites indicates a need for multivariable metabolite models, instead of single metabolite models, to elucidate important metabolic profiles among patients with COPD. One approach to multivariable metabolite modeling entails creating a score. Metabolomic scores are useful in predicting a variety of chronic diseases and disease risk markers, including incident coronary heart disease [7,8], weight gain [9], and type 2 diabetes [10]. The methods used to develop these scores range from least absolute shrinkage and selection operator (LASSO) to random forest modeling. These scores can also sometimes include clinical markers. However, a metabolomic score has not been developed for COPD. Pinto-Plata et al. evaluated a panel of untargeted metabolomics using random forest and support vector machines to classify controls, surviving patients, and nonsurviving patients with COPD, but they did not develop a score metric [11]. Furthermore, none of these studies used an independent population to validate the performance of a COPD score.

A challenge in developing scores is determining which variables to use, particularly when the number of variables is larger than the number of subjects and when there is collinearity among variables. As with LASSO, the adaptive LASSO (adaLASSO) model shrinks beta parameters to exactly 0 to drop unimportant metabolites, and additionally, adaLASSO adds individual weights for each variable to control bias in estimators, which allows for more consistent variable selection. The coefficients from the adaLASSO can then be used as weights to develop a final score and are interpretable in terms of the linear relationship between the metabolite and the outcome. In this analysis, we use adaLASSO to develop separate metabolomic severity scores (metSSs) for two important clinical manifestations of COPD—airflow limitation and emphysema—using two independent cohorts. Since airflow limitation is relatively easy and inexpensive to assess with spirometry, the severity scores may demonstrate the mechanistic associations between the clinical manifestations and metabolic disturbances.

## 2. Results

### 2.1. Demographic Characteristics

Demographics and clinical characteristics for both the training and validation cohorts are presented in Table 1. Cohorts show significant differences in all characteristics except for sex and BMI. The SPIROMICS (training) cohort had a greater proportion of self-identified Black/African American participants (18.8% vs. 8.5%, Chi-squared *p*-value < 0.001), and a higher percentage of current smokers (33.5%, vs. 23.9%, Chi-squared *p*-value < 0.001). However, the COPDGene (validation) cohort had more participants with Global Initiative for Chronic Obstructive Lung Disease (GOLD) stage 4 (5.6% vs. 2.3%, *p*-value < 0.001), a lower postbronchodilator FEV_1_ (2.2 vs. 2.3 L, *t*-test *p*-value = 0.003), and lower MESA-adjusted lung density (g/L) (81.7 vs. 86.1 g/L, *t*-test *p*-value < 0.001) indicating more severe COPD.

### 2.2. Adaptive LASSO Results

**Figure 1:**
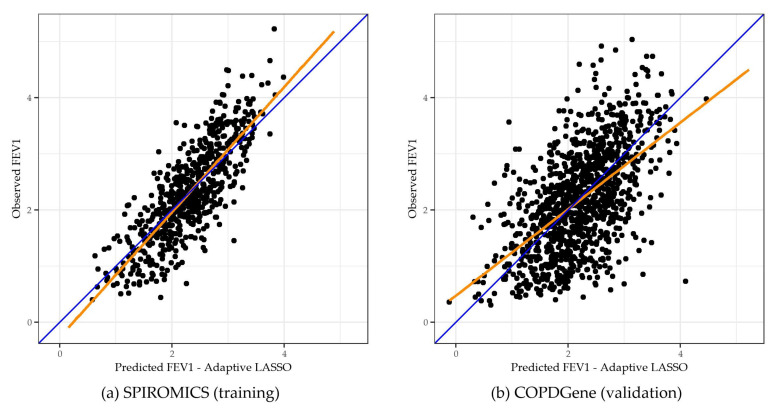
metSS Predicted vs. Observed FEV_1_ for the Training (**a**) and Validation (**b**) Cohorts Adaptive LASSO-based metSS prediction of FEV1. Each point represents an individual’s predicted and observed FEV_1_. The blue identity line denotes a perfect prediction of FEV_1_. The orange line denotes the observed FEV_1_ regressed on the metSS-predicted FEV_1_.

For FEV_1_, the adaLASSO procedure selected a total of 132 metabolites. The top 25 metabolites are shown in Table 2 (all metabolites and weights are shown in Table S1). Additionally, to depict the association of the adaLASSO-selected metabolites and FEV_1_, scatterplots of the four largest-coefficient metabolites are shown in Figure S1. In the validation data set, the metSS-only model explained 31.7% of the variability in FEV_1_. The combined metSS and covariate model explained 53.4% of the variability, which was more than the covariate-only model (46.4%) (*p* < 0.001). In the training data set, the metSS-only model explained almost 1.5 times the variability in FEV_1_ compared with the covariate-only model (64.8% vs. 42.1%) (Table 3). When combined, the clinical covariates and metSS explained 4.1% more variability compared with the metSS-only model (*p* < 0.001) in the training cohort. Mean squared error (MSE) showed similar patterns in the error for each model. Figure 1 shows the relationship between the metSS-predicted FEV_1_ values and the observed FEV_1_ values in the training and validation data sets. The figure shows a bias in the predicted scores with predictions for the highest and lowest FEV_1_ values being under- and over-predicted, respectively.

Linear regression models of covariates-only, metSS-only, and metSS with covariates for MESA-adjusted lung density showed a similar pattern to the corresponding models for FEV_1_ (Table S2). The adaLASSO model selected 129 metabolites for MESA-adjusted lung density. In the validation data set, the metSS explained 17.4% of the variability; however, the combined metSS and covariate model outperformed the covariate-only model (adjusted R^2^: 42.2% vs. 38.2%). In the training data set, the metSS-only models explained 59% variability in MESA-adjusted lung density, while combined metSS and covariate models explained 63% of the variability, similar patterns were seen for MSE in both the validation and training cohorts.

### 2.3. Pathway Analysis

**Figure 2:**
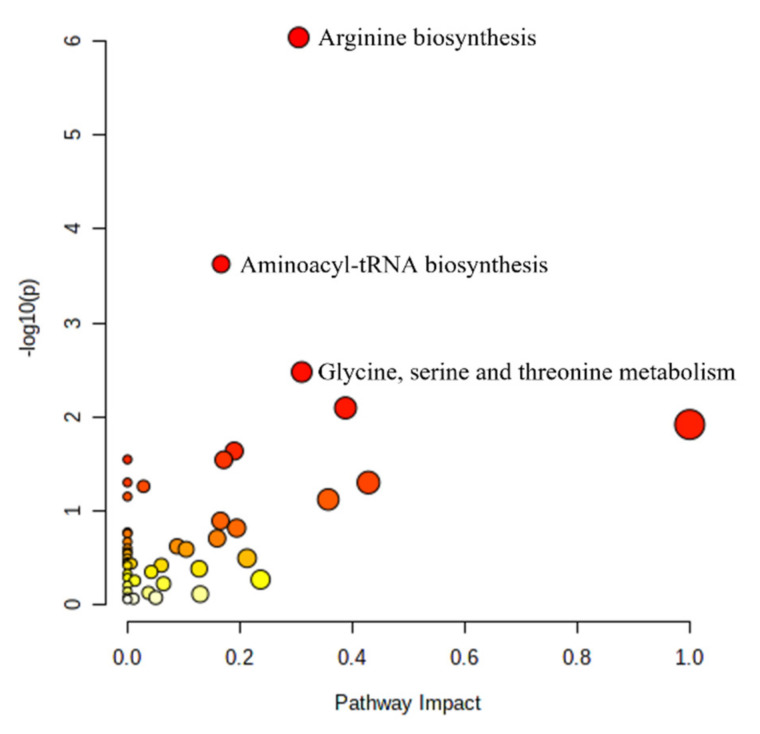
Pathway Analysis for FEV_1_-annotated pathways indicate pathways with FDR < 0.1. The colors for each pathway refer to the −log_10_(*p*) of the over-representation raw *p*-value (unadjusted for multiple comparisons) with red to yellow indicating a higher to lower −log_10_(*p*) values. The pathway impact represents the importance of the matched metabolites normalized by the importance of all metabolites in the pathway.

For FEV_1_, metabolites in three KEGG pathways were identified by the MetaboAnalyst 5.0 pathway analysis tool as over-represented (hypergeometric FDR < 0.10): arginine biosynthesis; aminoacyl-tRNA biosynthesis; and glycine, serine, and threonine metabolism, as shown in Figure 2. The specific metabolites in each of the significant pathways and their coefficients (weights) in the adaLASSO are shown in Table 4.

### 2.4. Sensitivity Analysis

As a sensitivity analysis, the training and validation data sets were exchanged and metSSs were developed in the COPDGene cohort and validated in the SPIROMICS cohort. Results are presented in Tables S3 and S4. In brief, the results were similar with the combined metSS and covariate models having the highest explained variability for both FEV_1_ and MESA-adjusted lung density in both the training and validation data sets; however, almost double the number of metabolites were chosen by the adaLASSO procedure. Additionally, to correct for the bias depicted in Figure 1, the adaLASSO procedure was run with the highest and lowest quintiles of the training data weighted by a factor of 5. This incurs a higher penalty in the procedure for incorrectly predicting these subjects. A smaller number of metabolites were selected by adaLASSO using higher weights for extreme values. The linear models using the metSS from the extreme value weighted adaLASSO produced similar results to the original model, with the combined metSS and covariate model explaining the most variability in FEV_1_ and MESA-adjusted lung density; however, the metSS-only models underperformed compared with the original analysis.

## 3. Discussion

This is the first publication of a metabolomic severity score for a respiratory disease. The major advantage of the metSS is that, similar to a genetic risk score, it combines the predictive power of many variables that individually, typically, explain only a small percentage (<5%) of the variability of a phenotype. For instance, in the absence of clinical covariates, our metSS was able to explain 32% of the variability in the independent validation cohort, similar to the SNP-based polygenic risk scores developed on hundreds of thousands of individuals [12]. Indeed, when clinical covariates (sex, age, height, race/ethnicity, BMI, smoking status, smoking pack-years, and clinical center) were added to the metSS, there was a 7% increase in explanation of variance over the covariate-only model. These findings support a key role of the blood metabolome in understanding COPD, as the metabolome reflects various physiological processes important in COPD pathogenesis, from immune regulation and energy homeostasis to protein synthesis/degradation and skeletal muscle dysfunction.

We considered two approaches to generating a metSS: with clinical covariates and without clinical covariates. The major advantage of a metSS without clinical covariates is that it is a standalone blood test and does not need separate interpretation based on age, sex, race, etc. An alternative would be to develop a score that included those covariates; however, that would limit the application of metSS to only subjects who have all covariates measured. Thus, using only metabolites in the adaLASSO procedure to derive a severity score allows for a broadly useful score for the population.

Studies have used a variety of methods to develop risk scores for different diseases. For this metSS, our goal was to create a parsimonious model for FEV_1_ using only metabolite data. Since the number of metabolites exceeds the sample size (*p* > *n*), and metabolites can be highly correlated, adaLASSO was our preferred method for variable selection. As with LASSO, adaLASSO performs both variable selection and effect estimation simultaneously. However, adaLASSO has an advantage over LASSO in that it uses individual weights for each variable to reduce the bias in large coefficients found in LASSO [13]. For this analysis, the inverse of the absolute value of ridge regression coefficients were used as individual penalties for each metabolite, which allows for correlated metabolites to be included in the final model. Additionally, adaLASSO has been shown to select the true subset of variables and estimate the weight of the true variables as if only the true variables were included in the model in simulated datasets, which is called the oracle property [13,14]. One drawback to adaLASSO is that it lacks the ability to model nonlinear effects. However, metabolites selected by adaLASSO are interpretable in the same way as effects from multivariable regression models. Other methods such as random forests and support vector regression may be able to model nonlinear effects, but they can be difficult to interpret.

As adaLASSO has the ability to select only signal metabolomic features (i.e., the oracle property), it is worth investigating which metabolomic features are selected in the metSS. Approximately 70% of selected metabolites consisted of amino acid and lipid super pathway metabolites, split evenly between the two categories. The five metabolites with the strongest weights were vanillylmandelate (VMA), N1-methyladenosine, glutamine, 2-hydroxypalmitate, and choline phosphate. While this is the first report of these metabolites and lung function or COPD, other metabolites selected by adaLASSO have been reported to be associated with COPD (Table S1). For instance, dimethylarginine and, specifically, asymmetric dimethylarginine (ADMA) results in a “functionally relevant shift” in l-arginine breakdown and has been associated with airflow obstruction [15]. Sphingomyelin has been associated with progression of percent emphysema and with worsening lung function [4,16]. Finally, 12,13 DiHOME has been associated with sex-specific effects with increased levels found in female smokers compared with female non-smokers [17].

The KEGG pathways that were over-represented in the metabolites selected in the adaLASSO procedure were arginine biosynthesis; aminoacyl-tRNA biosynthesis; and glycine, serine, and threonine metabolism. Glutamine and arginine, important amino acids in both the arginine biosynthesis and aminoacyl-t-RNA biosynthesis pathways, were inversely associated with FEV_1_. These findings are concordant with prior studies that found serum glutamine to be elevated in individuals with COPD compared with controls, and both serum glutamine and arginine to be elevated specifically in individuals with GOLD 4 COPD compared with controls [18,19].

AdaLASSO selection of dimethylarginine (DMA) further supports dysregulation of the arginine pathway in COPD. DMA is produced when methylated arginine residue proteins are degraded. DMA can be stimulated by hypoxia and is important in inflammation because it inhibits nitric oxide synthases (NOS) [20]. Our findings are supported by several smaller publications. In a study of 44 COPD patients and 30 healthy subjects, DMA was higher in patients with COPD [21]. These findings were supported by another study of 58 COPD patients and 30 healthy subjects [20]. In another study of 23 moderate-to-severe COPD patients and 19 healthy older controls, whole-body arginine was higher in COPD patients and related to de novo arginine production [22]. Additionally, the arginine pathway was also implicated in smoke-mediated emphysema in mice through its role in oxidant/antioxidant balance [23]. However, in a study of 25 COPD patients and 21 controls, a negative association was found between arginine and COPD status [24], while in another study comparing healthy smokers and smokers with COPD, Naz et al. found differences by sex with COPD women having lower ratios of arginine/(citrulline + ornithine) and higher ratios of asymmetric (ADMA) and symmetric dimethylarginine (SDMA) to arginine compared with healthy female smokers, but no difference in men [25]. KEGG analysis also identified aminoacyl-tRNA biosynthesis and glycine, serine, and threonine pathway overrepresentation in the metSS, which suggests disturbances in general amino acid metabolism. Aminoacyl-tRNAs are vital for protein synthesis and have been associated with oxidative stress [5,6,26]. Similarly, multiple studies have found enrichment of the glycine, serine, and threonine metabolism pathways, which have been associated with COPD exacerbation severity [4,27]. The reason for amino acid metabolism dysfunction in COPD is unclear but has been speculated to be a result of systemic inflammation and skeletal muscle energy metabolism dysfunction [28].

There are several limitations to our metSS approach. For instance, our metSS was developed in a population enriched with COPD, which could limit the generalizability and the deconvolution of those metabolomic pathways related to COPD progression versus those that regulate lung development and variability in the general population. This is supported by the overall low, but not negligible, overlap of metabolites associated with FEV_1_ between our analysis and those involving general population cohorts that included a significant proportion of individuals who never smoked and/or who had normal FEV_1_ [5,6]. Beyond disease progression versus lung-development-related metabolites, differences in methodological approaches (adaLASSO vs. multivariable regression models) between analyses might also explain these results and warrant further investigation. Additionally, the severity score was developed with a cross-sectional sample and the findings may not to apply to the longitudinal progression of COPD within an individual. The utility of the metSS should be tested with a longitudinal sample to determine the viability of the metSS in predicting progression of COPD. We chose the SPIROMICS data as our training set, even though it was smaller than COPDGene, as the population was more racial/ethnically diverse, had a higher proportion of current smokers and had less severe disease status, which should improve the utility of the score in undiagnosed populations and in early COPD patients where disease identification and prevention is most relevant. Age is also an important factor for the metabolome; the cohorts used to derive and validate the severity score were older adults, which again limits the generalizability of the score to younger populations. Finally, due to the low cost and accessibility of spirometry, the metSS, currently, should be used in research settings to better understand the pathobiology of COPD rather than a clinical tool.

In summary, we show that we can use adaLASSO to generate a metSS, similar to polygenic risk scores, which is highly associated with the variability of FEV_1_ in two independent cohorts of individuals with a smoking history and with or at risk for COPD. The FEV_1_ metSS is significantly enriched in amino acid pathways (particularly, arginine metabolism), suggesting the importance of these pathways in COPD pathogenesis. Further work in younger subjects without disease who have multiple long-term spirometry assessments should be undertaken to assess whether a metSS can predict disease development or progression.

## 4. Materials and Methods

### 4.1. Study Populations

#### 4.1.1. Training Cohort

The Subpopulations and Intermediate Outcome Measures in COPD Study (SPIROMICS) (ClinicalTrials.gov Identifier: NCT01969344) provided metabolomic data for training in the development of our metSS. In brief, this study recruited 2771 participants between 40–80 years old with at least 20 pack-years of smoking and 202 participants who were never smokers; 73% of participants self-identified as non-Hispanic white. Of participants returning for their 5–7 year follow-up visit, the first 649 were selected for metabolomic profiling of baseline fasting blood samples. Details of the cohort are provided elsewhere [29,30]. Data from 648 participants were used in this analysis, as one subject was excluded. Median standard deviation scores (z-scores) were calculated across metabolites at the subject level. Subjects with aggregate metabolite median z-scores > 3 SD from the mean of the cohort were removed.

#### 4.1.2. Validation Cohort

The Genetic Epidemiology of COPD (COPDGene) (ClinicalTrials.gov Identifier: NCT00608764), another NIH-sponsored multicenter cohort, provided metabolomic data for validation of the metSS. Details of the COPDGene study are provided elsewhere [31]. In brief, this study enrolled 10,198 non-Hispanic white and African American participants between 40–80 years with at least 10 pack-years of smoking and no exacerbations for more than 30 days, and 465 individuals with no smoking history. At the in-person, 5-year visit, 1136 participants from the National Jewish Health and University of Iowa clinical centers participated in an ancillary study in which nonfasting blood samples were collected and processed for metabolomic profiling [29,31,32]. Data from 1125 participants were used in this analysis and six subjects were excluded based on median standard deviation score (z-scores), as described above, and another five had missing values of covariates.

Informed consent was obtained from all subjects involved in the study.

### 4.2. Data and Definitions

#### 4.2.1. Clinical Data and Definitions

COPD severity and interindividual differences are best measured by FEV_1_ and emphysema, respectively; due to this heterogeneity, both postbronchodilator forced expiratory volume in 1 s (FEV_1_) and quantitative emphysema were used to generate separate metSSs. Emphysema was quantified using MESA-adjusted lung density from a computed tomography scan of the chest and adjusted according to reference equations from the Multi-Ethnic Study of Atherosclerosis [33]. Clinical covariates included age at time of visit, sex, height, self-identified race/ethnicity, BMI (kg/m^2^), current smoking status, smoking pack-years, and clinical center. Additionally, for MESA-adjusted lung density, scanner model was included as a covariate. We tested for differences between the two cohorts using t-tests for continuous variables, and chi-squared tests and Fisher’s Exact test for categorical variables.

#### 4.2.2. Metabolomic Profiling and Processing

Plasma samples from both cohorts were profiled using Metabolon (Durham, NC, USA) Global Metabolomics Platform, as described previously, although profiling for each cohort occurred approximately 1 year apart [34,35,36]. Metabolite values were batch normalized, within each study, by dividing by the median metabolite value for each metabolite within a batch. After batch normalization, metabolite PCs showed a significant reduction in association with batch, so no further normalization was needed [32].

Metabolites were excluded if >80% of samples were missing values (COPDGene: 149; SPIROMICS: 197). For metabolites missing in 20–80% of samples, a present/absent (1/0) indicator variable was created and used for all analyses (COPDGene: 248, SPIROMICS: 192). For metabolites missing in <20% of samples, missing values were imputed with k-nearest neighbor imputation (kNN; k = 10) using the R package ‘impute’ (COPDGene: 995; SPRIOMICS: 785). In the SPIROMICS study, a total of 1174 metabolites were characterized by Metabolon, whereas 1392 metabolites were characterized in the COPDGene study. For the 7 tobacco metabolites identified by Metabolon and common between studies, 1 (nicotine) was excluded since >80% of samples were missing values, and the other six were converted to present/absent indicator variables.

### 4.3. Analysis

#### 4.3.1. Statistical and Bioinformatics Analysis

Continuous metabolite values were natural log-transformed for all analyses. Of the 946 metabolites identified in both studies, 73 had <20% missing samples in one study and 20–80% missing in the other, and 111 had at least one cohort with ≥80% missing samples; 673 had kNN imputed missing values in both cohorts and 89 were dichotomized to present/absent variables in both studies, resulting in 762 metabolites used to develop the metSSs.

Separate adaptive least absolute shrinkage and selection operator (adaLASSO) analyses were performed to select metabolites and their weights for the metSSs for FEV_1_ and MESA-adjusted lung density. Inverted absolute value ridge regression estimates were used as initial weights for adaLASSO, and a 10-fold cross validation was performed in the training data set to obtain the penalty parameter (λ), which corresponded to the lowest mean-squared error across the folds. The adaLASSO procedure did not include clinical covariates. The metSSs were created with the coefficients and set of metabolites selected by the adaLASSO procedure.

Separate linear regression models were run to assess the variability explained (adjusted R^2^ using the Wherry formula) by (a) the clinical covariates, (b) the metSS, and (c) the metSS in concert with clinical covariates associated with FEV_1_ and MESA-adjusted lung density. Both training and validation data sets were modeled using linear regression. Additionally, mean squared error (MSE) was calculated to assess fit. Sensitivity analyses were performed to test the effect of switching the training and validation cohorts and by adding a higher penalty to errors in the highest and lowest quintiles of the training cohort.

#### 4.3.2. Pathway Analysis

Pathway analysis was conducted using MetaboAnalyst 5.0 web server (accessed 2 November 2021) for KEGG pathways. Details on pathway analysis were previously published [37]. In brief, pathway analysis used a hypergeometric test to determine over-representation of pathways based on metabolites selected by adaLASSO and, additionally, used measures of metabolite centrality, including relative betweenness centrality and out-degree centrality, to calculate importance in the pathway and determine pathway impact. Pathway analysis was conducted on metabolites selected by the adaLASSO procedure.

#### 4.3.3. Software

The statistical software R Version 4.0.2 was used for all analyses. The R packages glmnet and stats were used for adaLASSO and linear regression models, respectively. The MetaboAnalyst webserver (https://www.metaboanalyst.ca/, accessed on 2 November 2021) was used for pathway analysis. Analysis code can be accessed on GitHub at https://github.com/sunigodbole/netco-metRS, accessed on 2 November 2021.

## Figures and Tables

**Table 1:** Training and Validation Data Characteristics.

Characteristic	SPIROMICS (Training) (*n* = 648)	COPDGene (Validation) (*n* = 1120)	*p*
Age (yrs): mean (sd)	63.2 (8.89)	67.3 (8.82)	<0.001
Sex: *n* (%)			
Male	349 (53.9)	562 (50.2)	0.149
Female	299 (46.1)	558 (49.8)	
Race/Ethnicity: *n* (%)			
Non-Hispanic, White	469 (72.4)	1025 (91.5)	<0.001
Black/African American	122 (18.8)	95 (8.5)	
Other	57 (8.8)	0 (0)	
BMI (kg/m^2^)	28.4 (5.22)	28.8 (6.14)	0.206
Spirometry category ^†^: *n* (%)			
PRISm	11 (1.7)	101 (9.1)	<0.001
GOLD 0	261 (40.3)	505 (45.7)	
GOLD 1	113 (17.5)	114 (10.3)	
GOLD 2	181 (28)	208 (18.8)	
GOLD 3	66 (10.2)	115 (10.4)	
GOLD 4	15 (2.3)	62 (5.6)	
Smoking Status: *n* (%)			
Never Smoker	57 (8.9)	65 (5.8)	<0.001
Former Smoker	369 (57.6)	787 (70.3)	
Current Smoker	215 (33.5)	268 (23.9)	
Smoking pack-yrs: mean (sd)	45.1 (30.94)	42.4 (26.18)	0.048
Postbronchodilator FEV_1_ (L): mean (sd)	2.32 (0.84)	2.19 (0.91)	0.003
Postbronchodilator FEV_1_/FVC: mean (sd)	0.65 (0.15)	0.67 (0.15)	0.008
Postbronchodilator FEV_1_ percent predicted: mean (sd)	80.7 (23.5)	79.3 (26.5)	0.297
Percent Emphysema ^‡^: mean (sd)	5.79 (8.08)	6.66 (9.8)	0.058
MESA-adjusted lung density (g/L): mean (sd)	86.1 (24.4)	81.7 (22.6)	<0.001

*t*-tests used for continuous variables and Chi-squared/Fisher’s Exact for categorical variables. ^†^ PRISm (Preserved Ratio Impaired Spirometry) defined as postbronchodilator FEV1/FVC ≥ 0.7 and FEV1 % predicted < 80%; GOLD 0 defined as postbronchodilator FEV1/FVC ≥ 0.7 and FEV1 % predicted ≥ 80%; GOLD 1–4 defined as postbronchodilator FEV1/FVC < 0.7 and FEV1 % predicted ≥ 80% for GOLD 1, 50–80% for GOLD 2, 30–50% for GOLD 3, <30% for GOLD 4 ^‡^ measured as percent of lung voxels <−950 Hounsfield units.

**Table 2:** Top 25 metabolites for FEV_1_ from adaLASSO analysis, adaLASSO coefficient, and Super and Sub pathways annotated from Metabolon.

Metabolite	adaLASSO β	Super Pathway (Metabolon)	Sub Pathway (Metabolon)
vanillylmandelate (VMA)	−0.5654	Amino Acid	Tyrosine Metabolism
N1-methyladenosine	−0.3571	Nucleotide	Purine Metabolism, Adenine containing
Glutamine	−0.3414	Amino Acid	Glutamate Metabolism
2-hydroxypalmitate	−0.3264	Lipid	Fatty Acid, Monohydroxy
choline phosphate	0.2924	Lipid	Phospholipid Metabolism
1-palmitoyl-2-stearoyl-GPC (16:0/18:0)	0.2912	Lipid	Phosphatidylcholine (PC)
cerotoylcarnitine (C26) *	0.2859	Lipid	Fatty Acid Metabolism (Acyl Carnitine, Long Chain Saturated)
phenylalanine	−0.2675	Amino Acid	Phenylalanine Metabolism
dimethylarginine (SDMA + ADMA)	0.2646	Amino Acid	Urea cycle; Arginine and Proline Metabolism
myo-inositol	0.2568	Lipid	Inositol Metabolism
imidazole lactate	0.2561	Amino Acid	Histidine Metabolism
1-stearoyl-2-arachidonoyl-GPC (18:0/20:4)	−0.2456	Lipid	Phosphatidylcholine (PC)
N-acetylvaline	0.2374	Amino Acid	Leucine, Isoleucine and Valine Metabolism
taurine	−0.2325	Amino Acid	Methionine, Cysteine, SAM, and Taurine Metabolism
sulfate *	0.2325	Xenobiotics	Chemical
3-methyl-2-oxovalerate	−0.2206	Amino Acid	Leucine, Isoleucine, and Valine Metabolism
gamma-glutamylthreonine	0.2162	Peptide	Gamma-glutamyl Amino Acid
proline	0.2127	Amino Acid	Urea cycle; Arginine and Proline Metabolism
mannonate *	−0.2107	Xenobiotics	Food Component/Plant
retinol (vitamin A)	0.2103	Cofactors and Vitamins	Vitamin A Metabolism
sphingomyelin (d18:2/21:0, d16:2/23:0) *	−0.1919	Lipid	Sphingomyelins
N-acetylcarnosine	0.1825	Amino Acid	Histidine Metabolism
3beta-hydroxy-5-cholestenoate	0.1798	Lipid	Sterol
pimelate (C7-DC)	−0.1790	Lipid	Fatty Acid, Dicarboxylate

**Table 3:** Adjusted R^2^ and Mean Squared Error (MSE) to assess Linear Regression Models for postbronchodilator FEV_1_ (L).

	FEV_1_ (132 Metabolites)					
	Adjusted R^2^			MSE		
	Clinical Covariates ^1^ Only	metSS Only	metSS + Covariates ^1^	Clinical Covariates ^1^ Only	metSS Only	metSS + Covariates ^1^
SPIROMICS (training)	42.1	64.8	68.9	0.397	0.246	0.213
COPDGene (validation)	46.4	31.7	53.4	0.435	0.559	0.378

^1^ clinical covariates: sex, age, height, race/ethnicity, BMI, smoking status, smoking pack-years, and clinical site.

**Table 4:** Metabolites and adaLASSO Coefficients in Significant KEGG Pathways (FDR < 0.1).

KEGG Pathway	adaLASSO (β)
*Arginine Biosynthesis*	
glutamine	−0.34
arginine	−0.11
N-acetylglutamate	−0.10
citrulline	−0.10
alpha-ketoglutarate	−0.07
aspartate	0.07
fumarate	−0.06
*Aminoacyl-tRNA Biosynthesis*	
glutamine	−0.34
phenylalanine	−0.27
proline	0.21
threonine	−0.16
tyrosine	−0.12
arginine	−0.11
aspartate	0.07
serine	0.05
cysteine	0.05
*Glycine, serine, and threonine metabolism*	
threonine	−0.16
creatine	−0.12
serine	0.05
cysteine	0.05
sarcosine	−0.03
choline	0.02

## Data Availability

Clinical data with definitions can be found on dbGaP for COPDGene (phs000179.v6.p2) and SPIROMICS (phs001119.v1.p1). For COPDGene and SPIROMICS, the following clinical data were used: COPDGene_P1P2_All_Visit_29Sep2018 and V5_DERV_INV1_200127, respectively. COPDGene and SPIROMICS metabolomic data are available at the NIH Common Fund’s National Metabolomics Data Repository (NMDR) website, the MetabolomicsWorkbench, https://www.metabolomicsworkbench.org (accessed on 7 March 2022) (Study IDs ST002089 and ST002088, respectively).

## Supplementary Materials

The following supporting information can be downloaded at https://www.mdpi.com/article/10.3390/metabo12050368/s1, Figure S1: Scatterplots of the FEV1 postbronchodilator (L) and four strongest effect metabolites from the adaLASSO selection in the training and validation cohort.; Table S1: All metabolites selected by the adaLASSO procedure, the adaLASSO coefficient, super pathway, and sub pathway from Metabolon processing; Table S2: Adjusted R^2^ and MSE from Linear Regression Model for MESA-adjusted Lung Density; Table S3: Sensitivity Analysis: Adjusted R^2^ and MSE from Linear Regression Models for FEV_1_; Table S4: Sensitivity Analysis: Adjusted R^2^ and MSE from Linear Regression Models for MESA-adjusted Lung Density.

## References

[1] Global Initiative for Chronic Obstructive Lung Disease—GOLD (2021). Global Strategy for the Diagnosis, Management, and Prevention of Chronic Obstructive Pulmonary Disease (2022 Report).

[2] Mannino D.M., Gagnon R.C., Petty T.L., Lydick E. (2000). Obstructive lung disease and low lung function in adults in the United States: Data from the National Health and Nutrition Examination Survey, 1988–1994. Arch. Intern. Med..

[3] World Health Organization (2021). Chronic Obstructive Pulmonary Disease (COPD). https://www.who.int/news-room/fact-sheets/detail/chronic-obstructive-pulmonary-disease-(copd).

[4] Cruickshank-Quinn C., Jacobson S., Hughes G., Powell R.L., Petrache I., Kechris K., Bowler R., Reisdorph N. (2018). Metabolomics and transcriptomics pathway approach reveals outcome-specific perturbations in COPD. Sci. Rep..

[5] Yu B., Flexeder C., McGarrah R.W., Wyss A., Morrison A.C., North K.E., Boerwinkle E., Kastenmüller G., Gieger C., Suhre K. (2019). Metabolomics Identifies Novel Blood Biomarkers of Pulmonary Function and COPD in the General Population. Metabolities.

[6] Kelly R.S., Stewart I.D., Bayne H., Kachroo P., Spiro A., Vokonas P., Sparrow D., Weiss S.T., Knihtilä H.M., Litonjua A.A. (2021). Metabolomic differences in lung function metrics: Evidence from two cohorts. Thorax.

[7] Vaarhorst A.A., Verhoeven A., Weller C.M., Böhringer S., Göraler S., Meissner A., Deelder A.M., Henneman P., Gorgels A.P., van den Brandt P. (2014). A metabolomic profile is associated with the risk of incident coronary heart disease. Am. Heart J..

[8] Wang Z., Zhu C., Nambi V., Morrison A.C., Folsom A.R., Ballantyne C.M., Boerwinkle E., Yu B. (2019). Metabolomic Pattern Predicts Incident Coronary Heart Disease: Findings from the Atherosclerosis Risk in Communities Study. Arterioscler. Thromb. Vasc. Biol..

[9] Geidenstam N., Hsu Y.-H.H., Astley C.M., Mercader J.M., Ridderstråle M., Gonzalez M.E., Gonzalez C., Hirschorn J.N., Salem R.M. (2019). Using metabolite profiling to construct and validate a metabolite risk score for predicting future weight gain. PLoS ONE.

[10] Floegel A., Stefan N., Yu Z., Mühlenbruch K., Drogan D., Joost H.-G., Fritsche A., Häring H.-U., de Angelis M.H., Peters A. (2013). Identification of Serum Metabolites Associated with Risk of Type 2 Diabetes Using a Targeted Metabolomic Approach. Diabetes.

[11] Pinto-Plata V., Casanova C., Divo M., Tesfaigzi Y., Calhoun V., Sui J., Polverino F., Priolo C., Petersen H., de Torres J.P. (2019). Plasma metabolomics and clinical predictors of survival differences in COPD patients. Respir. Res..

[12] Moll M., Sakornsakolpat P., Shrine N., Hobbs B., DeMeo D.L., John C., Guyatt A.L., McGeachie M.J., Gharib S.A., Obeidat M. (2020). Chronic obstructive pulmonary disease and related phenotypes: Polygenic risk scores in population-based and case-control cohorts. Lancet Respir. Med..

[13] Huang J., Ma S., Zhang C.-H. (2008). Adaptive LASSO for Sparse High-Dimensional Regression Models. Stat. Sin..

[14] Zou H. (2006). The Adaptive Lasso and Its Oracle Properties. J. Am. Stat. Assoc..

[15] Scott J.A., Duongh M., Young A.W., Subbarao P., Gauvreau G.M., Grasemann H. (2014). Asymmetric Dimethylarginine in Chronic Obstructive Pulmonary Disease (ADMA in COPD). Int. J. Mol. Sci..

[16] Ahmed F.S., Jiang X.-C., Schwartz J.E., Hoffman E.A., Yeboah J., Shea S., Burkart C.M., Barr R.G. (2014). Plasma sphingomyelin and longitudinal change in percent emphysema on CT. The MESA Lung study. Biomarkers.

[17] Wheelock C., Balgoma D., Grunewald J., Eklund A., Skold M., Wheelock A. Lipid mediator levels evidence gender-specific increases in bronchoalveolar lavage fluid of COPD patients relative to healthy smokers. Proceedings of the European Respiratory Society Annual Congress.

[18] Ubhi B.K., Cheng K.K., Dong J., Janowitz T., Jodrell D., Tal-Singer R., MacNee W., Lomas D.A., Riley J.H., Griffin J.L. (2012). Targeted metabolomics identifies perturbations in amino acid metabolism that sub-classify patients with COPD. Mol. BioSyst..

[19] Ubhi B.K., Riley J.H., Shaw P.A., Lomas D.A., Tal-Singer R., MacNee W., Griffin J.L., Connor S.C. (2012). Metabolic profiling detects biomarkers of protein degradation in COPD patients. Eur. Respir. J..

[20] Aydin M., Altintas N., Mutlu L.C., Bilir B., Oran M., Tulubas F., Topçu B., Tayfur I., Kucukyalcin V., Kaplan G. (2017). Asymmetric dimethylarginine contributes to airway nitric oxide deficiency in patients with COPD. Clin. Respir. J..

[21] Ruzsics I., Nagy L., Keki S., Sarosi V., Illes B., Illes Z., Horvath I., Bogar L., Molnar T. (2016). L-Arginine Pathway in COPD Patients with Acute Exacerbation: A New Potential Biomarker. COPD J. Chronic Obstr. Pulm. Dis..

[22] Jonker R., Deutz N.E., Erbland M.L., Anderson P.J., Engelen M.P. (2016). Alterations in whole-body arginine metabolism in chronic obstructive pulmonary disease. Am. J. Clin. Nutr..

[23] Santos Valença S., Rueff-Barroso C.R., Alves Pimenta W., Correa Melo A., Tiscoski Nesi R., Santos Silva M.A., Porto L.C. (2011). L-NAME and L-arginine differentially ameliorate cigarette smoke-induced emphysema in mice. Pulm. Pharmacol. Ther..

[24] Kilk K., Aug A., Ottas A., Soomets U., Altraja S., Altraja A. (2018). Phenotyping of Chronic Obstructive Pulmonary Disease Based on the Integration of Metabolomes and Clinical Characteristics. Int. J. Mol. Sci..

[25] Naz S., Kolmert J., Yang M., Reinke S.N., Kamleh M.A., Snowden S., Heyder T., Levänen B., Erle D.J., Sköld C.M. (2017). Metabolomics analysis identifies sex-associated metabotypes of oxidative stress and the autotaxin–lysoPA axis in COPD. Eur. Respir. J..

[26] Tang Y., Chen Z., Fang Z., Zhao J., Zhou Y., Tang C. (2021). Multi-omics study on biomarker and pathway discovery of chronic ob-structive pulmonary disease. J. Breath Res..

[27] Ran N., Pang Z., Gu Y., Pan H., Zuo X., Guan X., Yuan Y., Wang Z., Guo Y., Cui Z. (2019). An updated overview of metabolomic profile changes in chronic obstructive pulmonary disease. Metabolites.

[28] Engelen M.P.K.J., Wouters E.F.M., Deutz N.E.P., Does J.D., Schols A.M.W.J. (2001). Effects of Exercise on Amino Acid Metabolism in Patients with Chronic Obstructive Pulmonary Disease. Am. J. Respir. Crit. Care Med..

[29] Gillenwater L., Kechris K., Pratte K., DeMeo D., Bowler R. (2020). Metabolomic Profiling Reveals Sex Specific Associations with COPD and Emphysema. Metabolites.

[30] Couper D., LaVange L.M., Han M., Barr R.G., Bleecker E., Hoffman E., Kanner R., Kleerup E., Martinez F.J., Woodruff P.G. (2013). Design of the Subpopulations and Intermediate Outcomes in COPD Study (SPIROMICS). Thorax.

[31] Regan E.A., Hokanson J.E., Murphy J.R., Make B., Lynch D.A., Beaty T.H., Curran-Everett D., Silverman E.K., Crapo J.D. (2010). Genetic Epidemiology of COPD (COPDGene) Study Design. COPD J. Chronic Obstr. Pulm. Dis..

[32] Gillenwater L.A., Pratte K.A., Hobbs B.D., Cho M.H., Zhuang Y., Halper-Stromberg E., Cruickshank-Quinn C., Reisdorph N., Petrache I., Labaki W.W. (2020). Plasma Metabolomic Signatures of Chronic Obstructive Pulmonary Disease and the Impact of Genetic Variants on Phenotype-Driven Modules. Netw. Syst. Med..

[33] Hoffman E., Ahmed F.S., Baumhauer H., Budoff M., Carr J., Kronmal R., Reddy S., Barr R.G. (2014). Variation in the Percent of Emphysema-like Lung in a Healthy, Nonsmoking Multiethnic Sample. The MESA Lung Study. Ann. Am. Thorac. Soc..

[34] DeHaven C.D., Evans A.M., Dai H., Lawton K.A. (2010). Organization of GC/MS and LC/MS metabolomics data into chemical libraries. J. Cheminform..

[35] Evans A.M., DeHaven C.D., Barrett T., Mitchell M., Milgram E. (2009). Integrated, nontargeted ultrahigh performance liquid chromatography/electrospray ionization tandem mass spectrometry platform for the identification and relative quantification of the small-molecule complement of biological systems. Anal. Chem..

[36] Miller M.J., Kennedy A.D., Eckhart A.D., Burrage L.C., Wulff J.E., Miller L.A., Milburn M.V., Ryals J.A., Beaudet A.L., Sun Q. (2015). Untargeted metabolomic analysis for the clinical screening of inborn errors of metabolism. J. Inherit. Metab. Dis..

[37] Xia J., Wishart D.S. (2010). MetPA: A web-based metabolomics tool for pathway analysis and visualization. Bioinformatics.

