# Supplementary material for: A Metabolomic Severity Score for Airflow Obstruction and Emphysema
Source: Metabolites. 2022 Apr 19;12(5):368. doi: 10.3390/metabo12050368 (PMC9143560; doi:10.3390/metabo12050368)
Supplement: Supplementary file 1 [file metabolites-12-00368-s001.zip › metabolites-1663701-supplementary.pdf]

Table S1: All metabolites selected with the adaLASSO procedure, the adaLASSO coefficient, and super pathway and sub pathway annotation from Metabolon.

Metabolite	adaLASSO β	Super Pathway (Metabolon)	Sub Pathway (Metabolon)
vanillylmandelate (VMA)	−0.5654	Amino Acid	Tyrosine Metabolism
N1−methyladenosine	−0.3571	Nucleotide	Purine Metabolism, Adenine containing
glutamine	−0.3414	Amino Acid	Glutamate Metabolism
2−hydroxypalmitate	−0.3264	Lipid	Fatty Acid, Monohydroxy
choline phosphate	0.2924	Lipid	Phospholipid Metabolism
1−palmitoyl−2−stearoyl−GPC (16:0/18:0)	0.2912	Lipid	Phosphatidylcholine (PC)
cerotoylcarnitine (C26)*	0.2859	Lipid	Fatty Acid Metabolism (Acyl Carnitine, Long Chain Saturated)
phenylalanine	−0.2675	Amino Acid	Phenylalanine Metabolism
dimethylarginine (SDMA + ADMA)	0.2646	Amino Acid	Urea cycle; Arginine and Proline Metabolism
myo−inositol	0.2568	Lipid	Inositol Metabolism
imidazole lactate	0.2561	Amino Acid	Histidine Metabolism
1−stearoyl−2−arachidonoyl−GPC (18:0/20:4)	−0.2456	Lipid	Phosphatidylcholine (PC)
N−acetylvaline	0.2374	Amino Acid	Leucine, Isoleucine and Valine Metabolism
taurine	−0.2325	Amino Acid	Methionine, Cysteine, SAM and Taurine Metabolism
sulfate*	0.2325	Xenobiotics	Chemical
3−methyl−2−oxovalerate	−0.2206	Amino Acid	Leucine, Isoleucine and Valine Metabolism
gamma−glutamylthreonine	0.2162	Peptide	Gamma−glutamyl Amino Acid
proline	0.2127	Amino Acid	Urea cycle; Arginine and Proline Metabolism
mannonate*	−0.2107	Xenobiotics	Food Component/Plant
retinol (vitamin A)	0.2103	Cofactors and Vitamins	Vitamin A Metabolism
sphingomyelin (d18:2/21:0, d16:2/23:0)*	−0.1919	Lipid	Sphingomyelins
N−acetylcarnosine	0.1825	Amino Acid	Histidine Metabolism
3beta−hydroxy−5−cholestenoate	0.1798	Lipid	Sterol
pimelate (C7−DC)	−0.1790	Lipid	Fatty Acid, Dicarboxylate
3−hydroxy−2−ethylpropionate	0.1707	Amino Acid	Leucine, Isoleucine and Valine Metabolism
12,13−DiHOME	0.1642	Lipid	Fatty Acid, Dihydroxy
threonine	−0.1623	Amino Acid	Glycine, Serine and Threonine Metabolism
2'−O−methyluridine	−0.1592	Nucleotide	Pyrimidine Metabolism, Uracil containing
glycosyl−N−palmitoyl−sphingosine (d18:1/16:0)	−0.1482	Lipid	Hexosylceramides (HCER)
1−(1−enyl−palmitoyl)−GPC (P−16:0)*	0.1409	Lipid	Lysoplasmalogen
thyroxine	−0.1408	Amino Acid	Tyrosine Metabolism
N−acetylalanine	0.1276	Amino Acid	Alanine and Aspartate Metabolism
salicyluric glucuronide*	−0.1253	Xenobiotics	Drug − Analgesics, Anesthetics
5−hydroxyhexanoate	−0.1220	Lipid	Fatty Acid, Monohydroxy
carnitine	−0.1200	Lipid	Carnitine Metabolism
tyrosine	−0.1188	Amino Acid	Tyrosine Metabolism
creatine	−0.1155	Amino Acid	Creatine Metabolism
cysteinylglycine	0.1146	Amino Acid	Glutathione Metabolism
ergothioneine	0.1142	Xenobiotics	Food Component/Plant
alpha−tocopherol	−0.1141	Cofactors and Vitamins	Tocopherol Metabolism
arginine	−0.1121	Amino Acid	Urea cycle; Arginine and Proline Metabolism
glucuronide of C12H22O4 (1)*	−0.1109	Partially Characterized Molecules	Partially Characterized Molecules
alpha−hydroxyisocaproate	0.1095	Amino Acid	Leucine, Isoleucine and Valine Metabolism

Metabolite	adaLASSO β	Super Pathway (Metabolon)	Sub Pathway (Metabolon)
hydroxyasparagine**	−0.1038	Amino Acid	Alanine and Aspartate Metabolism
L−urobilin	0.1023	Cofactors and Vitamins	Hemoglobin and Porphyrin Metabolism
N−acetylglutamate	−0.0973	Amino Acid	Glutamate Metabolism
citrulline	−0.0967	Amino Acid	Urea cycle; Arginine and Proline Metabolism
biliverdin	0.0967	Cofactors and Vitamins	Hemoglobin and Porphyrin Metabolism
5alpha−androstan−3alpha,17beta−diol disulfate	0.0933	Lipid	Androgenic Steroids
7alpha−hydroxy−3−oxo−4−cholestenoate (7−Hoca)	−0.0927	Lipid	Sterol
2−palmitoyl−GPC (16:0)*	0.0904	Lipid	Lysophospholipid
3−ureidopropionate	0.0875	Nucleotide	Pyrimidine Metabolism, Uracil containing
nisinate (24:6n3)	0.0855	Lipid	Long Chain Polyunsaturated Fatty Acid (n3 and n6)
1−linoleoyl−2−arachidonoyl−GPC (18:2/20:4n6)*	−0.0846	Lipid	Phosphatidylcholine (PC)
1−(1−enyl−palmitoyl)−2−palmitoleoyl−GPC (P−16:0/16:1)*	−0.0829	Lipid	Plasmalogen
acisoga	−0.0813	Amino Acid	Polyamine Metabolism
alpha−ketoglutaramate*	0.0775	Amino Acid	Glutamate Metabolism
alpha−ketoglutarate	−0.0749	Energy	TCA Cycle
alpha−hydroxycaproate	−0.0744	Lipid	Fatty Acid, Monohydroxy
erucate (22:1n9)	−0.0737	Lipid	Long Chain Monounsaturated Fatty Acid
1−(1−enyl−palmitoyl)−2−oleoyl−GPC (P−16:0/18:1)*	−0.0717	Lipid	Plasmalogen
aspartate	0.0696	Amino Acid	Alanine and Aspartate Metabolism
pregnenediol sulfate (C21H34O5S)*	0.0679	Lipid	Pregnenolone Steroids
N−stearoyl−sphingadienine (d18:2/18:0)*	−0.0668	Lipid	Ceramides
carboxyethyl−GABA	0.0641	Amino Acid	Glutamate Metabolism
fumarate	−0.0624	Energy	TCA Cycle
alpha−CEHC sulfate	−0.0622	Cofactors and Vitamins	Tocopherol Metabolism
gamma−glutamylleucine	0.0618	Peptide	Gamma−glutamyl Amino Acid
cis−4−decenoylcarnitine (C10:1)	−0.0577	Lipid	Fatty Acid Metabolism (Acyl Carnitine, Monounsaturated)
2−aminobutyrate	0.0571	Amino Acid	Glutathione Metabolism
prolylglycine	−0.0557	Peptide	Dipeptide
dihydroferulic acid sulfate	−0.0549	Xenobiotics	Food Component/Plant
serine	0.0528	Amino Acid	Glycine, Serine and Threonine Metabolism
lactate	−0.0485	Carbohydrate	Glycolysis, Gluconeogenesis, and Pyruvate Metabolism
N−acetylputrescine	−0.0484	Amino Acid	Polyamine Metabolism
N−acetylglycine	0.0483	Amino Acid	Glycine, Serine and Threonine Metabolism
cysteine	0.0471	Amino Acid	Methionine, Cysteine, SAM and Taurine Metabolism
octanoylcarnitine (C8)	−0.0455	Lipid	Fatty Acid Metabolism (Acyl Carnitine, Medium Chain)
adenosine 5'−monophosphate (AMP)	−0.0440	Nucleotide	Purine Metabolism, Adenine containing
vanillactate	−0.0436	Amino Acid	Tyrosine Metabolism
lactosyl−N−palmitoyl−sphingosine (d18:1/16:0)	0.0434	Lipid	Lactosylceramides (LCER)
3−methoxytyramine sulfate	−0.0428	Amino Acid	Tyrosine Metabolism
4−hydroxyglutamate	−0.0399	Amino Acid	Glutamate Metabolism
acesulfame	0.0311	Xenobiotics	Food Component/Plant
3−ethylcatechol sulfate (2)	0.0302	Xenobiotics	Food Component/Plant

Metabolite	adaLASSO β	Super Pathway (Metabolon)	Sub Pathway (Metabolon)
arachidonoylcarnitine (C20:4)	0.0301	Lipid	Fatty Acid Metabolism (Acyl Carnitine, Polyunsaturated)
N,N,N−trimethyl−5−aminovalerate	−0.0300	Amino Acid	Lysine Metabolism
eicosanedioate (C20−DC)	0.0298	Lipid	Fatty Acid, Dicarboxylate
bilirubin (Z,Z)	0.0295	Cofactors and Vitamins	Hemoglobin and Porphyrin Metabolism
sarcosine	−0.0293	Amino Acid	Glycine, Serine and Threonine Metabolism
valerate (5:0)	−0.0290	Lipid	Short Chain Fatty Acid
1−palmitoyl−2−palmitoleoyl−GPC (16:0/16:1)*	−0.0289	Lipid	Phosphatidylcholine (PC)
picolinate	−0.0284	Amino Acid	Tryptophan Metabolism
isobutyrylcarnitine (C4)	−0.0280	Amino Acid	Leucine, Isoleucine and Valine Metabolism
umbelliferone sulfate	0.0280	Xenobiotics	Food Component/Plant
glycerophosphoethanolamine	−0.0269	Lipid	Phospholipid Metabolism
sulfate of piperine metabolite C16H19NO3 (3)*	−0.0268	Xenobiotics	Food Component/Plant
1−(1−enyl−palmitoyl)−2−linoleoyl−GPC (P−16:0/18:2)*	0.0234	Lipid	Plasmalogen
1−stearoyl−2−docosahexaenoyl−GPC (18:0/22:6)	−0.0232	Lipid	Phosphatidylcholine (PC)
2−methylcitrate/homocitrate	0.0227	Energy	TCA Cycle
4−hydroxyphenylacetoylcarnitine	−0.0226	Amino Acid	Tyrosine Metabolism
indoleacetoylcarnitine*	−0.0226	Amino Acid	Tryptophan Metabolism
N−acetyl−isoputreanine	−0.0222	Amino Acid	Polyamine Metabolism
N−stearoyltaurine	−0.0207	Lipid	Endocannabinoid
N−acetylkynurenine (2)	0.0204	Amino Acid	Tryptophan Metabolism
5,6−dihydrothymine	−0.0204	Nucleotide	Pyrimidine Metabolism, Thymine containing
sphingomyelin (d18:1/18:1, d18:2/18:0)	−0.0204	Lipid	Sphingomyelins
isoleucylglycine	0.0200	Peptide	Dipeptide
choline	0.0196	Lipid	Phospholipid Metabolism
I−urobilinogen	−0.0192	Cofactors and Vitamins	Hemoglobin and Porphyrin Metabolism
1−stearoyl−2−linoleoyl−GPC (18:0/18:2)*	−0.0181	Lipid	Phosphatidylcholine (PC)
glycerol	−0.0173	Lipid	Glycerolipid Metabolism
3−hydroxyoleoylcarnitine	−0.0173	Lipid	Fatty Acid Metabolism (Acyl Carnitine, Hydroxy)
glucuronide of C10H18O2 (7)*	0.0167	Partially Characterized Molecules	Partially Characterized Molecules
docosadioate (C22−DC)	0.0154	Lipid	Fatty Acid, Dicarboxylate
nicotinamide	0.0153	Cofactors and Vitamins	Nicotinate and Nicotinamide Metabolism
beta−cryptoxanthin	0.0139	Cofactors and Vitamins	Vitamin A Metabolism
thioproline	0.0121	Xenobiotics	Chemical
6−oxopiperidine−2−carboxylate	−0.0109	Amino Acid	Lysine Metabolism
N−acetylglucosamine/N−acetylgalactosamine	−0.0108	Carbohydrate	Aminosugar Metabolism
1−methyl−4−imidazoleacetate	−0.0106	Amino Acid	Histidine Metabolism
2−acetamidophenol sulfate	0.0094	Xenobiotics	Food Component/Plant
3−methylcytidine	0.0088	Nucleotide	Pyrimidine Metabolism, Cytidine containing
stearoyl ethanolamide	0.0078	Lipid	Endocannabinoid
N−acetylglucosaminylasparagine	−0.0072	Carbohydrate	Aminosugar Metabolism
1,7−dimethylurate	−0.0071	Xenobiotics	Xanthine Metabolism
N4−acetylcytidine	−0.0057	Nucleotide	Pyrimidine Metabolism, Cytidine containing
campesterol	−0.0033	Lipid	Sterol
2−oxoarginine*	−0.0027	Amino Acid	Urea cycle; Arginine and Proline Metabolism
homoarginine	0.0010	Amino Acid	Urea cycle; Arginine and Proline Metabolism

Metabolite	adaLASSO β	Super Pathway (Metabolon)	Sub Pathway (Metabolon)
heneicosapentaenoate (21:5n3)	0.0006	Lipid	Long Chain Polyunsaturated Fatty Acid (n3 and n6)
oleoyl−arachidonoyl−glycerol (18:1/20:4) [2]*	0.0003	Lipid	Diacylglycerol

Table S2: Adjusted R^2 and MSE from Linear Regression Model for MESA−adjusted Lung Density

	MESA−Adjusted Lung Density (129 Metabolites)					
	**Adjusted R^2**			**MSE**		
	Clinical Covariates[1] Only	metSS Only	metSS + Covariates[1]	Clinical Covariates[1] Only	metSS Only	metSS + Covariates[1]
SPIROMICS (training)	38.1	59.0	63.0	359.17	243.23	214.19
COPDGene (validation)	38.2	17.4	42.2	311.41	419.87	291.13

[1]clinical covariates: gender, age, race/ethnicity, BMI, Smoking Status, Smoking pack−years, clinical site & CT scanner model

Table S3: Sensitivity Analysis: Adjusted R^2 and MSE from Linear Regression Models for FEV_1

			**FEV_1**					
			**Adjusted R^2**			**Mean Squared Error**		
		# of Met selected	Clinical Covariates[1] Only	metSS Only	metSS + Covariates[1]	Clinical Covariates[1] Only	metSS Only	metSS + Covariates[1]
Exchanging the training & validation data	COPDGene (training)	206	46.4	63.7	69.0	0.435	0.297	0.251
	SPIROMICS (validation)		42.1	33.6	47.8	0.397	0.465	0.357
Weighting the highest & lowest quintiles of values	SPIROMICS (training)	114	42.1	58.3	65.2	0.397	0.292	0.238
	COPDGene (validation)		46.4	26.1	51.7	0.435	0.605	0.392

[1]clinical covariates: gender, age, height, race/ethnicity, BMI, Smoking Status, Smoking pack−years & clinical site
"# of Met selected" refers to the number of metabolites selected by the adaptive LASSO.
For high and low quintile weighting the extreme value were weight by a factor of 5 in the training data set.

Table S4: Sensitivity Analysis: Adjusted R^2 and MSE from Linear Regression Models for MESA−adjusted Lung Density

			**MESA−adjusted Lung Density**					
			**Adjusted R^2**			**MSE**		
		# of Met selected	Clinical Covariates[1] Only	metSS Only	metSS + Covariates[1]	Clinical Covariates[1] Only	metSS Only	metSS + Covariates[1]
Exchanging the training & validation data	COPDGene (training)	275	38.2	64.4	68.7	311.41	181.10	157.62
	SPIROMICS (validation)		38.1	19.3	40.6	359.17	478.95	344.20
Weighting the highest & lowest quintiles of values	SPIROMICS (training)	133	38.1	55.5	60.3	359.17	264.16	229.87
	COPDGene (validation)		38.2	15.6	41.6	311.41	428.80	293.99

[1]clinical covariates: gender, age, race/ethnicity, BMI, Smoking Status, Smoking pack−years, clinical site & CT scanner model
"# of Met selected" refers to the number of metabolites selected by the adaptive LASSO.
For high and low quintile weighting the extreme value were weight by a factor of 5 in the training data set.

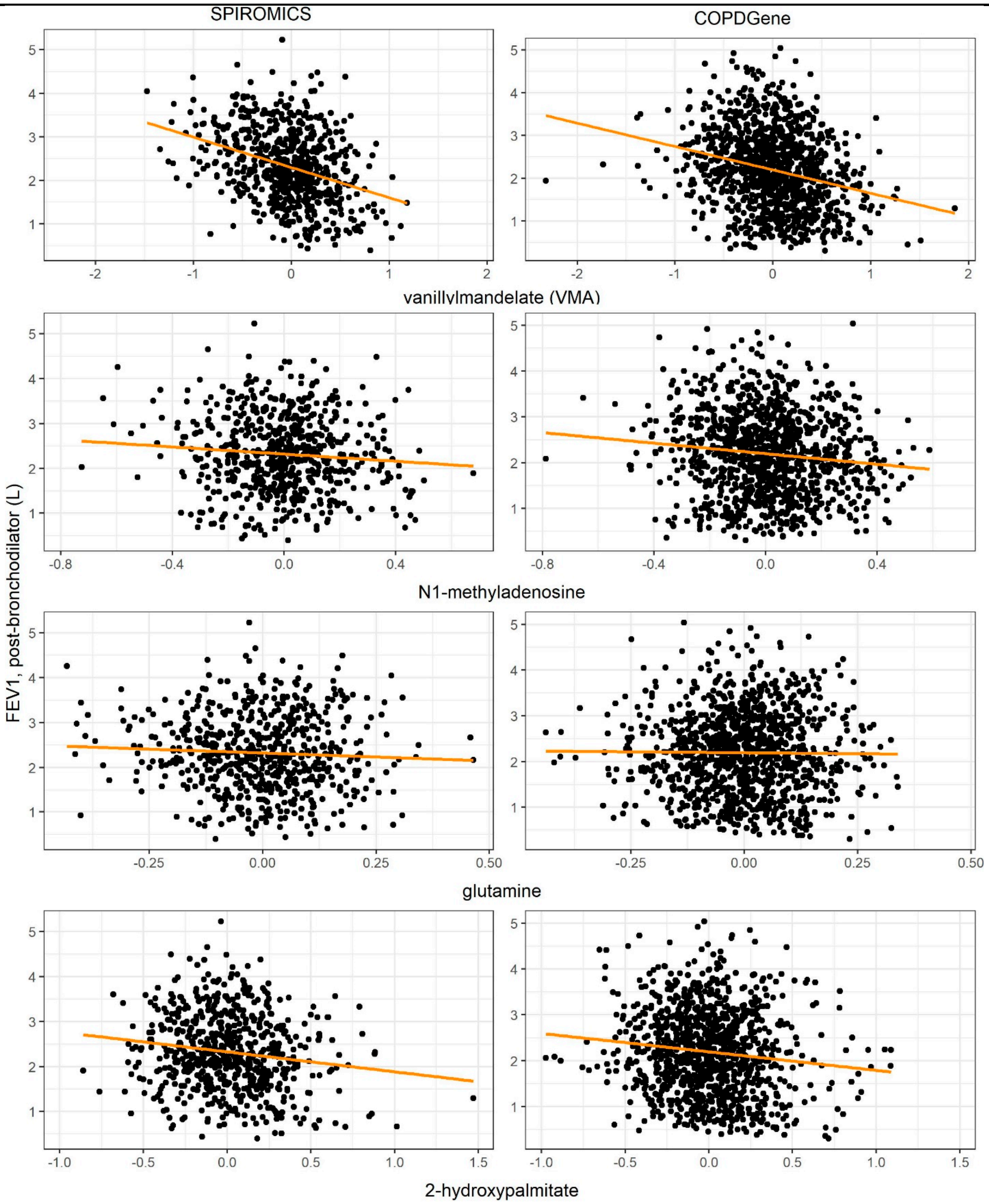

Figure S1: Scatterplots of FEV1 postbronchodilator (L) and 4 strongest effect metabolites from the adaLASSO model in the training and validation cohort. Orange line indicates the association between the single metabolite and FEV_1.
